# Early Spatiotemporal Patterns and Knee Kinematics during Level Walking in Individuals following Total Knee Arthroplasty

**DOI:** 10.1155/2017/7056469

**Published:** 2017-07-31

**Authors:** Xubo Wu, Lixi Chu, Lianbo Xiao, Yong He, Shuyun Jiang, Songbin Yang, Yijie Liu

**Affiliations:** ^1^School of Rehabilitation Science, Shanghai University of Traditional Chinese Medicine, Shanghai, China; ^2^Department of Orthopaedic Surgery, Guanghua Integrative Medicine Hospital, Shanghai, China; ^3^Gait Analysis Laboratory, Yueyang Hospital of Integrated Chinese and Western Medicine, Shanghai, China

## Abstract

**Purpose:**

With the aim of investigating the spatiotemporal features of early gait pattern and knee kinematics after total knee arthroplasty and analyzing the association between outcomes of gait analyses and knee kinematic parameters, the relationship between walking and dynamic knee deformity at the early period after total knee arthroplasty was assessed in this study.

**Methods:**

Eighteen patients including 14 women and 4 men who underwent total knee arthroplasty were analyzed using three-dimensional gait analysis system to observe gait parameters and values of maximum knee flexion angle (MKFA) during swing phase and knee flexion angle (KFA) and knee valgus angle (KVA) at midstance phase.

**Results:**

3D gait analysis showed that operated side exhibited significantly less total support time and single support time as well as significantly longer swing phase compared with the other side. During walking, the operated side had significantly smaller MKFA and greater KFA and KVA than the nonoperated side. There was moderate to significant correlation between gait pattern and the dynamic knee kinematics.

**Conclusion:**

The gait abnormality of patients after TKA was associated with inadequate flexion of knees at swing phase and insufficient extension at stance phase as well as increased range of valgus.

## 1. Introduction

Total knee arthroplasty (TKA) is performed to restore knee functions and relieve pain in some patients with severe osteoarthritis (OA). With the development of prosthesis design and surgical techniques, TKA has become a well-established treatment for managing end-stage symptomatic knee OA. However, not all patients following the operation obtain satisfactory outcomes. Studies indicated that only 70–89% of patients were satisfied with the surgery [1–3], while some patients suffered from pain, functional limitation, and even revision and indolent infection [4].

The satisfaction of patients is closely related to knee function after surgery and affected by a variety of postoperative complications. The way of improving the gait pattern and efficacy of patients after surgery is an important issue that challenges clinical professionals. More and more studies recommended early follow-up and monitoring of functional recovery [4, 5]. The way of identifying early specific indicators of some disorders in the future still remains as a problem for surgeons and other healthcare givers.

Kinematic alignment in TKA pursues better anatomical alignment of knee prosthesis with the aim of promoting more physiological motion and concerns with implant survivorship and patellofemoral tracking [6, 7]. However, a recent study showed that small deviations from the static mechanical axis alignment in TKA did not appear to impact overall survivorship or complication rates at short-term follow-up [8]. Moreover, some studies demonstrated that knee kinematics during gait in TKA group still differed from those of healthy control group despite of improved clinical outcomes and spatiotemporal parameters [9, 10].

This study used three-dimensional (3D) gait analysis technology to investigate the kinematic features of knee undergoing operation and explore the association between parameters of knee kinematics and spatiotemporal patterns during walking in the early recovery period after TKA and search for typical indicators specific to walking function, hence providing theoretical basis for early knee rehabilitation intervention after TKA surgery.

## 2. Materials and Methods

### 2.1. Subjects

The detailed data of eighteen patients with TKA, including 14 women and 4 men is provided in Table 1. There were 10 cases with left affected knee and 8 cases with right affected knee. All 18 patients were confirmed with primary osteoarthritis according to the diagnostic standard of osteoarthritis defined by the American College of Rheumatology. Additional exclusion criteria for patients were previous surgery or pain in the back or lower extremity, neurologic diseases, rheumatism, leg length discrepancy > 1 cm, a body mass index above 33 kg/m^2^, a history of major trauma or injury to the knee, and/or knee surgery within 6 months.

The patients were subjected to continuous epidural anesthesia. The median incision was made on the anterior area of knee with the selection of medial approach beside patella. All patients received surface replacement with prosthesis (Depuy PFC Sigma) fixed by bone cement. None of the patients received surface replacement of patella. After surgery, patients were given antibiotics to prevent infection. Meanwhile, low molecular heparin was subcutaneously injected into abdominal wall to prevent deep venous thrombosis in lower limbs.

All patients had their surgeries in the same surgery department of a hospital, showed good outcome scores, and were satisfied with the procedure. The patients with passive knee flexion in the operated knee of ≥90° and a HSS (the Hospital for Special Surgery, USA) score > 40 were included.

### 2.2. Gait Analysis

**Figure 1:**
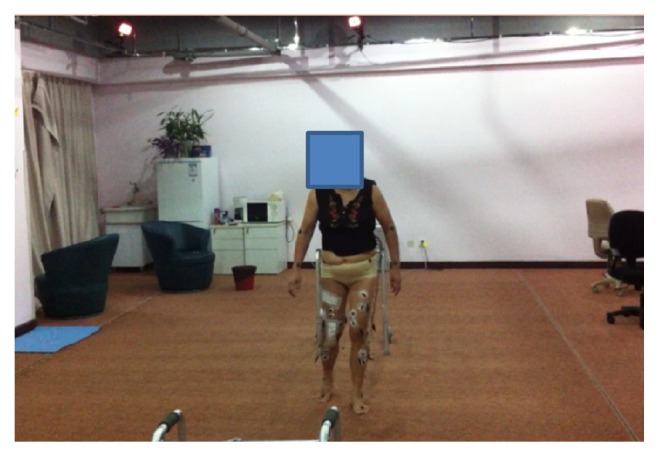
3D gait analysis technology.

Gait analysis was conducted for each patient on the 14th day after surgery. The subjects walked on flat ground at self-selected comfortable pace. As shown in Figure 1, the motions were captured using Motion Analysis System (Motion Analysis, USA), which had 12 infrared lenses at high speed including 9 Eagle-4 lenses and 3 Raptor-4 lenses. The ground reaction force of feet was tested by force platform (model OR6-7, AMTI, USA). The Cortex and OrthoTrack software (Motion Analysis, USA) were used for data analysis.

To get familiar with the environment, the patients entered the laboratory room of gait analysis 20 minutes prior to test. They were asked to wear swimwear or waistcoat and shorts to fully expose the locations of markers which were placed in accordance with the Helen Hayes gait model.

Before testing, the patients could practice a while to adapt to the environment and know the testing procedures. They were advised to walk back and forth at the most comfortable pace for one-way distance of about 5 meters between two walkers.

### 2.3. Data Extraction

The kinematic parameters of knee joint at sagittal plane and coronary plane were observed using 3D gait analysis technology. For the sagittal plane, the maximum knee flexion angle (MKFA) during swing phase and the knee flexion angle (KFA) at midstance phase were observed. For the coronary plane, the knee valgus angle (KVA) at midstance phase was observed.

A typical gait cycle was selected in this study. The MKFA was selected when the operated lower limb was at swing phase. As regards the stance phase of the operated lower limb, the angles of knee flexion and extension, valgus, and varus were recorded during the midstance phase which was associated with the period of single support.

### 2.4. Statistical Analysis

The statistical analysis was performed using SPSS 22.0 statistical software. The measurement data was expressed as mean ± standard deviation (*x* ± *s*). The analysis of normal distribution was conducted using K-S test. The analysis of corresponding parameters between two lower limbs during a typical gait cycle was conducted using paired *t*-test. The relationship between different parameters and gaits was analyzed using the Pearson correlation analysis. The test level was set at *α* = 0.05.

## 3. Results

### 3.1. Spatiotemporal Parameters

Three-dimensional gait analysis showed that operated side had significantly less total support time (*P* = 0.017) and longer swing phase (*P* = 0.017) as well as lower single support time (*P* = 0.017) compared with the other side. These results are provided in Table 2.

### 3.2. Measurements of Knee Kinematic Parameters

**Figure 2:**
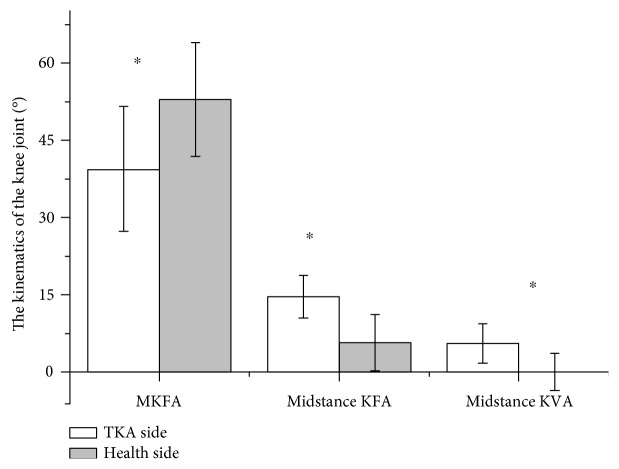
Comparison of joint angles between the operated and nonoperated knees (^∗^significance level refers to paired *t*-test comparing operated and nonoperated knee joint angles with *P* < 0.05. MKFA: maximum knee flexion angle; midstance KFA: knee flexion angle at midstance; midstance KVA: knee valgus angle at midstance).

During walking, the operated side had significantly smaller MKFA yet greater KFA and KVA at midstance phase than the nonoperated side. These results are provided in Table 3 and Figure 2.

### 3.3. Correlation between Kinematic Parameters of Knee and Parameters of Gait Analysis

MKFA of the operated knee during swing phase was positively correlated with ipsilateral average cadence (*r* = 0.636, *P* = 0.021). KFA of the operated knee at midstance phase was associated with the single support time of the same lower limb (*r* = −0.671, *P* = 0.038) negatively and the total support time of the other lower limb (*r* = 0.671, *P* = 0.038) positively. KVA of the operated knee at midstance phase was negatively associated with step frequencies of the other lower limb (*r* = −0.486, *P* = 0.041) and the painful lower limb (*r* = −0.597, *P* = 0.036). The results are shown in Table 4.

## 4. Discussion

The gait analysis was conducted in 18 patients on the 14th day after TKA. The results indicated that when patients walked at a comfortable speed, the operated lower limb exhibited significantly less single support time and total support time than nonoperated side. In addition to gait analysis parameters, this study also observed the kinematic parameters of the knee during walking, including MKFA at swing phase and KFA and KVA at midstance phase.

Although TKA surgery improved the static force lines of knee joint, only 20% of patients after a TKA exhibited a normal biphasic flexion-extension moment of the knee during level walking [3]. The limitation of knee flexion and extension after surgery is an important factor that impacts gait. Our study observed that inadequate extension of operated knee at midstance phase was about 14°, which caused shorter stance phase of operated limb than nonoperated limb (Table 2) and inadequate forward propulsion of body weight and further resulted in lower walking efficiency. Recent studies demonstrated that flexion of operated knee at stance phase was a core indicator that impacted the quality of gait [1, 11, 12], and knee flexion range in stance was the most important variable in discriminating between patients with TKA and controls [11].The study of Li et al. [13] noted that this was mostly due to inadequate muscle strength of quadriceps femoris and motor control disorder as well as lower moment of force during knee extension.

Table 4 shows that inadequate extension of operated lower limb at midstance phase led to prolonged stance phase of double support, featured by shorter single support time of the affected lower limb (*r* = −0.671) and longer total support time of the other lower limb (*r* = 0.671). The shorter stance phase of single support also caused shorter swing phase of the other side. Subsequently, inadequate swing phase caused reduced step length of the other side [14].

In addition to inadequate knee extension, increased valgus degree of operated knee at midstance phase was also observed in the study. There were significant statistical differences between the valgus of knees at stance phase of both legs (*P* < 0.01). The higher knee valgus of the operated knee during level walking was probably a compensatory mechanism, to avoid pain and provide energy for forward propulsion, and it might be associated with muscle control [15]. Some researchers noted that it might be associated with the type of implants. Renaud et al. compared both TKA designs and showed that Nexgen(TM) implant had significantly more flexion at the end of swing phase than Triathlon(TM) implant while at the midstance phase, Nexgen(TM) TKA exerted significantly more adduction [16]. Some authors actually found increased knee adduction moment during stance phase in TKA patients which stand in contrast to the present study [16, 17].

At the stance phase of the operated leg, there was an extension lag associated with the valgus of the knee, which caused changes in mechanical relationships between implant and polyethylene. The abnormal distribution of stress within implants was directly associated with wear of polyethylene pad, which might result in abnormal alignment of joint, implant loosening, pain in soft tissue surrounding the joint, and even shortening of implant survivorship.

The limitations of the study lay in the fact that during postoperative 2 weeks, the pain and swelling that affected the walking of the patients could be eased. Walking involved flexibility of joint and surrounding soft tissues, as well as accurate neuromuscular control and precise feedback by central nervous system [15, 18, 19]. For early patients with TKA, factors such as swelling of joint, pain, and lack of proprioception could impact the motion control on the knee joint, which influenced the motor performance of affected extremities [20–22].

## 5. Conclusion

There are significant abnormalities in early gait of patients after TKA. The gait abnormality is associated with inadequate flexion of knee at swing phase and insufficient extension at stance phase as well as increased range of valgus. However, TKA with early dynamic deviations in alignment interfered with gait poorly. Further prospective studies with longer term are needed to determine the precise indicators and mechanism of these kinematic parameters resulting in polyethylene wear, aseptic loosening, pain, and other complications.

## Figures and Tables

**Table 1:** General information of the patients.

	Minimum	Maximum	Mean	Standard deviation
Age (years)	48	79	64.50	9.45
Weight (kg)	46	89	68.22	11.13
Height (cm)	150	174	161.61	7.38
BMI (kg/m^2^)	18.66	29.76	25.99	2.85
HSS	42	77	64.06	8.93

**Table 2:** Comparisons of 3D gait parameters between two sides (*n* = 18).

	Operated side	Nonoperated side	Difference 95% CI	*P*
Step length avg (cm)	41.19 ± 12.42	39.67 ± 13.97	−1.570~4.834	0.297
Stride length avg (cm)	81.27 ± 25.20	81.00 ± 25.95	−0.726~1.710	0.406
Forward velocity avg (cm/s)	63.55 ± 25.21	63.62 ± 25.91	−0.847~1.127	0.768
Cadence avg (steps/min)	90.96 ± 16.27	91.31 ± 15.98	−0.951~0.580	0.616
Total support time (%)^∗^	65.07 ± 4.05	69.16 ± 8.48	−7.694~−0.874	0.017
Swing phase (%)^∗^	34.93 ± 4.05	30.84 ± 8.478	0.874~7.693	0.017
Initial double support time (%)	16.83 ± 6.25	17.74 ± 7.11	−4.882~2.481	0.501
Single support time (%)^∗^	30.84 ± 8.48	34.93 ± 4.05	−7.694~−0.874	0.017

^∗^Significant difference between two sides, *P* < 0.05.

**Table 3:** Knee kinematic parameters (degree) (*n* = 18).

	Operated side	Nonoperated side	Difference 95% CI	*P*
MKFA	39.43 ± 12.11	52.94 ± 11.05	−20.06~−6.97	0.000
Midstance KFA	14.71 ± 4.12	5.71 ± 5.51	5.62~12.37	0.000
Midstance KVA	5.55 ± 3.84	0.08 ± 3.58	3.26~7.68	0.000

MKFA: maximum knee flexion angle; midstance KFA: knee flexion angle at midstance; midstance KVA: knee valgus angle at midstance.

**Table 4:** Correlation between kinematic parameters of knee and parameters of gait analysis (*n* = 18).

	MKFA		Midstance KFA		Midstance KVA	
	*r*	*P*	*r*	*P*	*r*	*P*
Operated side cadence avg	0.636	0.021	−0.277	0.266	−0.486	0.041
Operated side single support time	0.412	0.089	−0.671	0.038	0.350	0.154
Nonoperated side cadence avg	0.418	0.084	−0.254	0.310	−0.597	0.036
Nonoperated side total support time	−0.412	0.089	0.671	0.038	−0.350	0.154

## References

[1] Dunbar M. J., Robertsson O., Ryd L., Lidgren L. (2001). Appropriate questionnaires for knee arthroplasty. Results of a survey of 3600 patients from The Swedish Knee Arthroplasty Registry. *Journal of Bone and Joint Surgery British Volume (London)*.

[2] Wylde V., Learmonth I., Potter A., Bettinson K., Lingard E. (2008). Patient-reported outcomes after fixed- versus mobile-bearing total knee replacement: a multi-centre randomised controlled trial using the Kinemax total knee replacement. *Journal of Bone and Joint Surgery British Volume (London)*.

[3] Bourne R. B., Chesworth B. M., Davis A. M., Mahomed N. N., Charron K. D. (2010). Patient satisfaction after total knee arthroplasty: who is satisfied and who is not?. *Clinical Orthopaedics and Related Research*.

[4] Hightower C. D., Hightower L. S., Tatman P. J., Morgan P. M., Gioe T., Singh J. A. (2016). How often is the office visit needed? Predicting total knee arthroplasty revision risk using pain/function scores. *BMC Health Services Research*.

[5] van Egmond J. C., Verburg H., Vehmeijer S. B., Mathijssen N. M. (2015). Early follow-up after primary total knee and total hip arthroplasty with rapid recovery: focus groups. *Acta Orthopaedica Belgica*.

[6] Fang D. M., Ritter M. A., Davis K. E. (2009). Coronal alignment in total knee arthroplasty: just how important is it?. *The Journal of Arthroplasty*.

[7] Ritter M. A., Davis K. E., Meding J. B., Pierson J. L., Berend M. E., Malinzak R. A. (2011). The effect of alignment and BMI on failure of total knee replacement. *The Journal of Bone and Joint Surgery American Volume*.

[8] Courtney P. M., Lee G. C. (2017). Early outcomes of kinematic alignment in primary total knee arthroplasty: a meta-analysis of the literature. *The Journal of Arthroplasty*.

[9] Bytyqi D., Shabani B., Cheze L., Neyret P., Lustig S. (2017). Does a third condyle TKA restore normal gait kinematics in varus knees? In vivo knee kinematic analysis. *Archives of Orthopaedic and Trauma Surgery*.

[10] Fenner V. U., Behrend H., Kuster M. S. (2017). Joint mechanics after total knee arthroplasty while descending stairs. *The Journal of Arthroplasty*.

[11] Rahman J., Tang Q., Monda M., Miles J., McCarthy I. (2015). Gait assessment as a functional outcome measure in total knee arthroplasty: a cross-sectional study. *BMC Musculoskeletal Disorders*.

[12] Ardestani M. M., Moazen M. (2016). How human gait responds to muscle impairment in total knee arthroplasty patients: muscular compensations and articular perturbations. *Journal of Biomechanics*.

[13] Li K., Ackland D. C., McClelland J. A. (2013). Trunk muscle action compensates for reduced quadriceps force during walking after total knee arthroplasty. *Gait & Posture*.

[14] Lee A., Park J., Lee S. (2015). Gait analysis of elderly women after total knee arthroplasty. *Journal of Physical Therapy Science*.

[15] Freisinger G. M., Hutter E. E., Lewis J. (2016). Relationships between varus-valgus laxity of the severely osteoarthritic knee and gait, instability, clinical performance, and function. *Journal of Orthopaedic Research*.

[16] Renaud A., Fuentes A., Hagemeister N., Lavigne M., Vendittoli P. A. (2016). Clinical and biomechanical evaluations of staged bilateral total knee arthroplasty patients with two different implant designs. *The Open Orthopaedics Journal*.

[17] Hatfield G. L., Stanish W. D., Hubley-Kozey C. L. (2015). Three-dimensional biomechanical gait characteristics at baseline are associated with progression to total knee arthroplasty. *Arthritis Care & Research (Hoboken)*.

[18] Neptune R. R., McGowan C. P. (2016). Muscle contributions to frontal plane angular momentum during walking. *Journal of Biomechanics*.

[19] Dubois G., Rouch P., Bonneau D., Gennisson J. L., Skalli W. (2016). Muscle parameters estimation based on biplanar radiography. *Computer Methods in Biomechanics and Biomedical Engineering*.

[20] Naili J. E., Iversen M. D., Esbjörnsson A. C. (2016). Deficits in functional performance and gait one year after total knee arthroplasty despite improved self-reported function. *Knee Surgery, Sports Traumatology, Arthroscopy*.

[21] Kang K. T., Koh Y. G., Son J. (2016). Measuring the effect of femoral malrotation on knee joint biomechanics for total knee arthroplasty using computational simulation. *Bone Joint Research*.

[22] Bonnefoy-Mazure A., Armand S., Sagawa Y., Suvà D., Miozzari H., Turcot K. (2017). Knee kinematic and clinical outcomes evolution before, 3 months, and 1 year after total knee arthroplasty. *The Journal of Arthroplasty*.

